# Does Hyoscine improve polyp detection rate during colonoscopy? Systematic Review & meta-analysis of randomized clinical trials

**DOI:** 10.1016/j.amsu.2018.10.020

**Published:** 2018-10-19

**Authors:** Khalid Hureibi, Pradip Abraham, Osama Alsunidar, Charles Evans, Kai Leong, Ling Wong

**Affiliations:** a University Hospital, Coventry, UK; b University of Science and Technology Hospital, Sana'a, Yemen

**Keywords:** Hyoscine Butylbrmoide, Buscopn, Colonoscopy, Polyp, Adenoma, Polyp detection rate, Adenoma detection rate

## Abstract

**Background/objective:**

Published studies have shown conflicting results regarding the benefit of Hyoscine Butylbrmoide use during colonoscopy in polyp and adenoma detection rates. This meta-analysis was conducted with the aim to summarize all available evidence.

**Methods:**

A literature search was carried out using PubMed, Ovid MEDLINE and the Cochrane Library database from inception to December 2017. Studies that compared the use of Hyoscine Butylbrmoide compared to placebo during colonoscopy were included. Pooled odds ratio and 95% confidence interval were calculated using Mantel-Haenszel fixed-effects model when there was no heterogeneity identified.

**Results:**

Of the 423 retrieved studies, eight met the eligibility criteria and were included in the analysis. There was no significant difference between the groups in terms of polyp and adenoma detection rates.

There was no significant difference between the Hyoscine and placebo groups in polyp detection rate (49.3% vs 48%, OR = 1.06, 95% CI: 0.90–1.23, P = 0.50). Adenoma detection rate was also not significantly different between the 2 groups 33.7% vs, 31%; OR = 1.13; 95%CI: 0.95–1.35; P = 0.16). No heterogeneity was observed (P = 0.65, I^2^ = 0%).

**Conclusion:**

This meta-analysis found no significant impact of Hyoscine on polyp and adenoma detection when used during colonoscopy.

## Highlights

• Buscopan has a controversial role in colonoscopy.
• More studies are needed that take into consideration endoscopists experience.
• The current evidence doesn't support the use of buscopan routinely during colonoscopy.

## 1 Introduction

Worldwide, colorectal cancer is the third commonest cancer and a leading cause of death from cancer [1]. More than 90% of colorectal cancer cases develop as a result of an adenoma-to-cancer sequence over many years [2], therefore, detecting and removing theses polyps early reduce incidence and mortality of colorectal cancer [3].

Colonoscopy is the gold standard in diagnosing and removing bowel polyps [4]. However, polyps can be overlooked during colonoscopy with a miss rate of 5%–32% [5].

Hyoscine Butylbrmoide is an antispasmodic drug that blocks the muscarinic receptors in the bowel, leading to a decrease in smooth muscle tone and motility [6], [7]. This may help to improve the colonscopic visualization of the bowel mucosa [8].

In a recent large study, the use of intravenous antispasmodic was associated with increased adenoma detection [9]. This observational study analyzed 31,088 colonoscopies from the English Bowel Cancer Screening Programme. It concluded that the use of intravenous hyoscine associated with a 30% higher adenoma detection.

However, there have been several RCTs (Randomized Controlled Trials) that showed conflicting results in the role of Hyoscine in polyp and adenoma detection rates [[10], [11], [12], [13], [14]]. Whilst two previous meta-analyses showed no significant difference between the intervention and placebo group [15] [16], a more recent meta-analysis concluded that Hyoscine may provide a “marginal improvement” in adenoma and polyp detection rates [17]. Since this last meta-analysis was published, a further three randomized controlled studies (n = 710) assessing the impact of hyoscine on polyp and adenoma detection rates have been published.

The aim of this meta-analysis was to investigate whether intravenous Hyoscine during colonoscopy had an effect of polyp and adenoma detection. RCTs that have assessed the role of intravenous hyoscine during colonoscopy were considered for inclusion with the aim of determining the effect of Hyoscine on polyp and adenoma detection rates.

## 2 Methods

This meta-analysis was undertaken and reported in accordance with the Preferred Reporting Items for Systematic Reviews and Meta-Analyses (PRISMA) statement [18].

### 2.1 Outcomes of interest

The primary and secondary outcomes are to assess the effect of Hyoscine Butylbrmoide on polyp detection rate when given intravenously during colonoscopy. The secondary outcome is to assess the same effect on adenoma detection rate.

### 2.2 Eligibility criteria

The following inclusion criteria were applied: Clinical prospective RCTs, comparing intravenous Hyoscine during colonoscopy with placebo. Outcomes assessed in the studies included: Polyp detection rate (PDR) and adenoma detection rate (ADR). Only publications in English language were included. There were no restrictions on dates published.

### 2.3 Search strategy

A systematic literature review was performed of PubMed, Ovid MEDLINE, EMBASE, Google Scholar and the Cochrane Library. All published studies up to 1st December 2017 were assessed. Hand searching of the literature references was also used during the same period. The following search terms were used; [title/abstract]: “Hyoscine N-butylbromide” OR “Buscopan” AND “polyp detection rate” OR “adenoma detection rate” OR “adenoma’ OR “polyp” OR “colonoscopy”. Abstracts were screened for relevance. Studies not published in English were excluded.

### 2.4 Study selection

The studies were extracted independently by KH, OA and PA according to the eligibility criteria. Any discrepancies were resolved by consensus discussion with CE. The following data were extracted: publication year and type (i.e., abstract or full article), study location, Number of patients and demographics, dose of Hyoscine and study endpoints.

### 2.5 Data collection & analysis

Data were extracted from the identified publications and recorded in Review Manager Version 5.3 (RevMan 5.3, The Nordic Cochrane Centre, The Cochrane Collaboration, Copenhagen, Denmark). Odds ratios (ORs) and their 95% confidence intervals (CIs) were calculated from the total number of patients and the number of events within each group. The PDR and ADR were the primary and secondary outcomes respectively.

The meta-analysis was performed using RevMan 5.3. Pooled odd ratios of PDR and ADR were calculated. Mantel-Haenszel fixed-effects model was used when there was no heterogeneity identified. Heterogeneity was assessed using χ^2^ and I^2^ tests (significant heterogeneity if p < 0.1 or I^2^ > 50%). Publication bias was assessed with the aid of funnel plots. Significance of the overall effect was determined using the z test. P values ≤ 0.05 were considered statistically significant.

### 2.6 Assessment of bias

The selected studies were assessed independently by KH, OA and PA for bias using the Cochrane Collaboration's tool for the assessment of bias [19]. Areas of disagreement were resolved by consensus discussion with CE.

## 3 Results

**Fig. 1:**
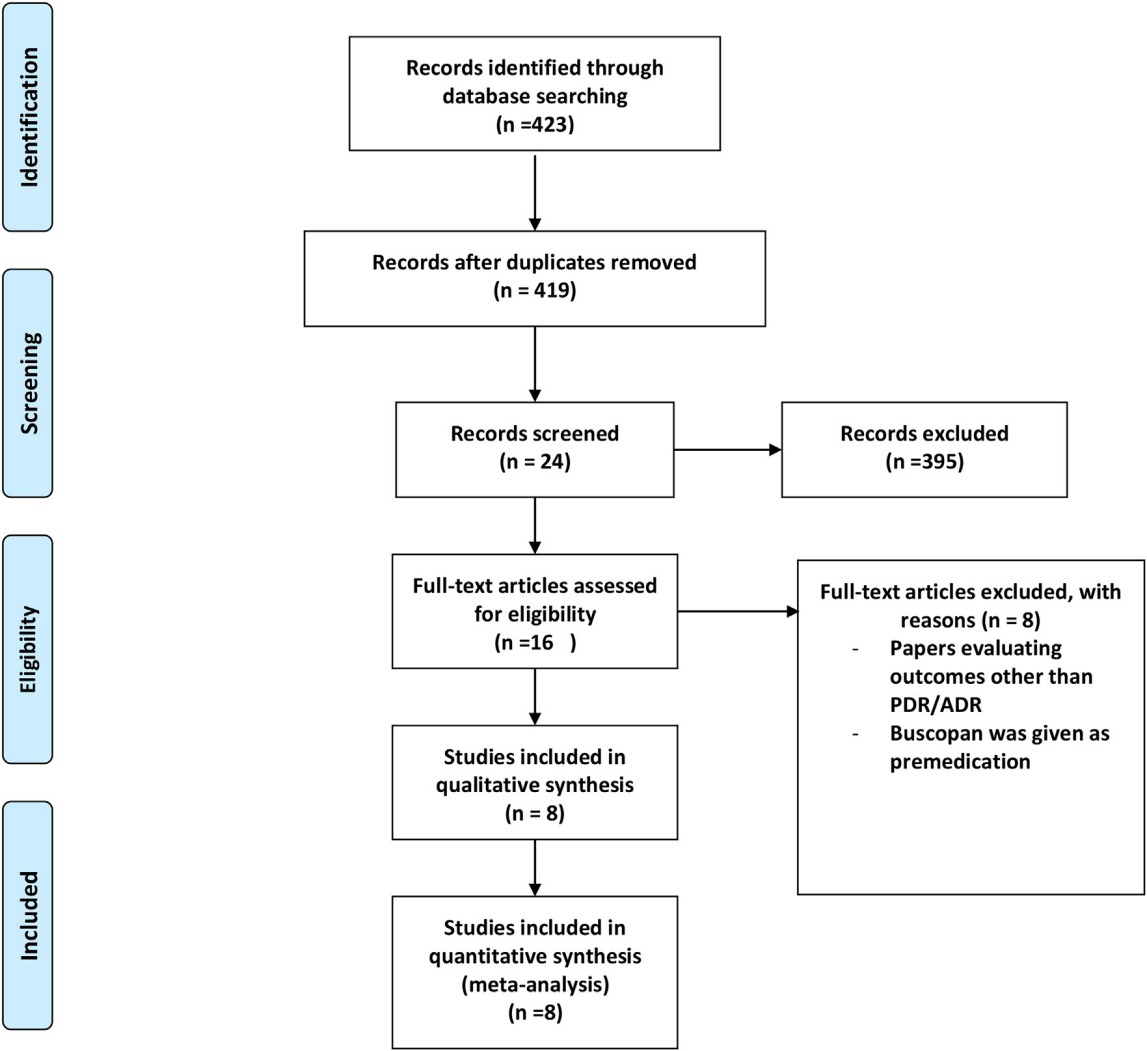
Prisma diagram.

The search resulted in 423 studies. After screening titles and abstracts, 16 papers were selected for full-text review. After applying the eligibility criteria, eight of these studies included in the meta-analysis [[10], [11], [12], [13], [14],[20], [21], [22]]. The PRISMA flow diagram is shown in Fig. 1. All of the selected studies were full text articles, except one study which was only available as an abstract [10]. Table 1 shows the characteristics of the included studies. The population of the reviewed studies included patients who were referred for elective diagnostic colonoscopy.

**Table 1:** Characteristics of the included studies.

Study (year)	Country	Type	Number of patients H/P	Age, years (mean) H/P	Male patients, (%) H/P	Good/excellent bowel preparation H/P (%)	PDR % H/P	Outcomes	Number of performers and experience
Byun et al., 2009	Korea	RCT	103/102	NA	NA	NA	45.6/39.2	PDR, ADR, polyp characteristics, procedure time, spasm score, vital signs, side effects	NA
Lee et al., 2010	Korea	RCT	58/58	∼59/58	46.6/39.7	94.8/93.1	34.5/25.9	PDR, polyp characteristics, modified colonic spasm scores, heart rate elevation	Single performer >1000 colonoscopies
de Brouwer et al., 2012	The Netherlands	RCT	340/334	∼62/61	45.9/52.7	7.9/8.1 (BBPS)	56/60	PDR, ADR, ALDR, 5% trimmed mean no. of polyps, mean withdrawal time	8 performers, at least 800 colonoscopies each
Corte et al., 2012	Australia	RCT	303/298	∼61/61	53.5/52.7	83.2/83.2	43.6/36.6	PDR, ADR	22 performers
Rondonotti et al., 2013	Italy	RCT	202/200	∼57/57	44.5/43.5	90/89	38.6/37.0	PDR, ADR, polyp morphology, change in AC, patient bloating perception, side effects of H	6 performers, at least 1500 colonoscopies each
Ristikankare et al., 2015	Finland	RCT	74/75	∼62/60	49/40	92/93	60.8/61.3	Polyps or tumours, ileal & caecal intubation times, total procedure time, patient tolerance, technical ease, heart rate elevation,	2 performers, several thousand colonoscopies each
Dinc et al., 2016	Turkey	RCT	60/61	52/55	51/55	95/90	28.3/29.5	Total procedure time, caecal intubation time, PDR, patient and endoscopist satisfaction score, heart rate elevation	6 performers
dos Santos et al., 2017	Brazil	RCT	220/220	NA	∼33/31	NA	65.9/64.6	PDR, ADR, adv-ADR, improvement in diagnostic accuracy of digital chromography,	Single performer >12,000 colonoscopies

H - Hyoscine, P - Placebo, PDR - polyp detection rate, ADR - adenoma detection rate, ALDR - advanced lesion detection rate, AC - abdominal circumference, RCT - randomized controlled trial, IBD - inflammatory bowel disease.

**Fig. 2:**
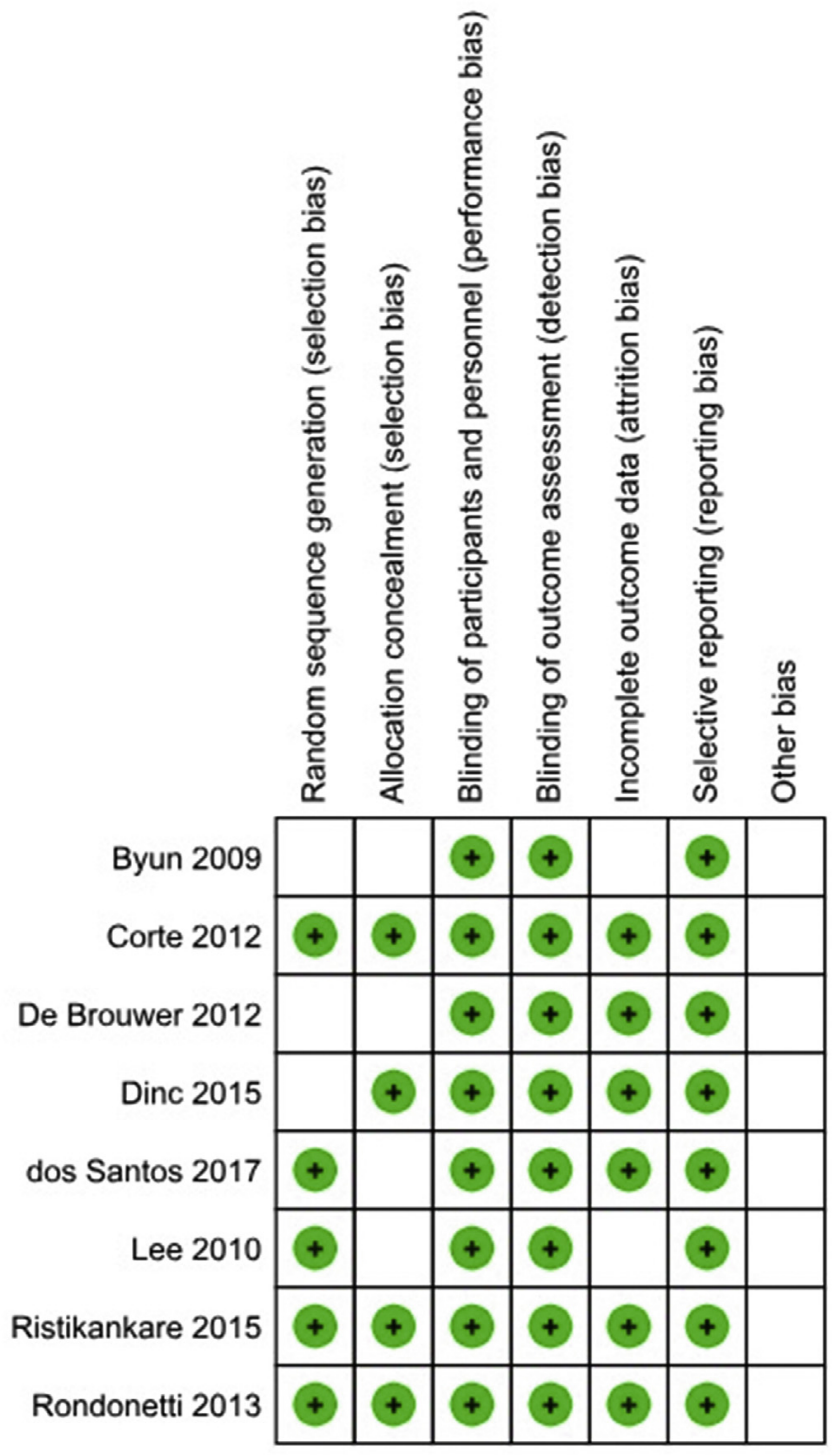
Assessment of bias.

2708 patients were included in this meta-analysis, including 1360 patients in the Hyoscine group and 1348 patients in the control group. The main characteristics of each study are shown in Table 1. The assessment of bias is shown in Fig. 2.

### 3.1 Polyp detection rate

**Fig. 3:**
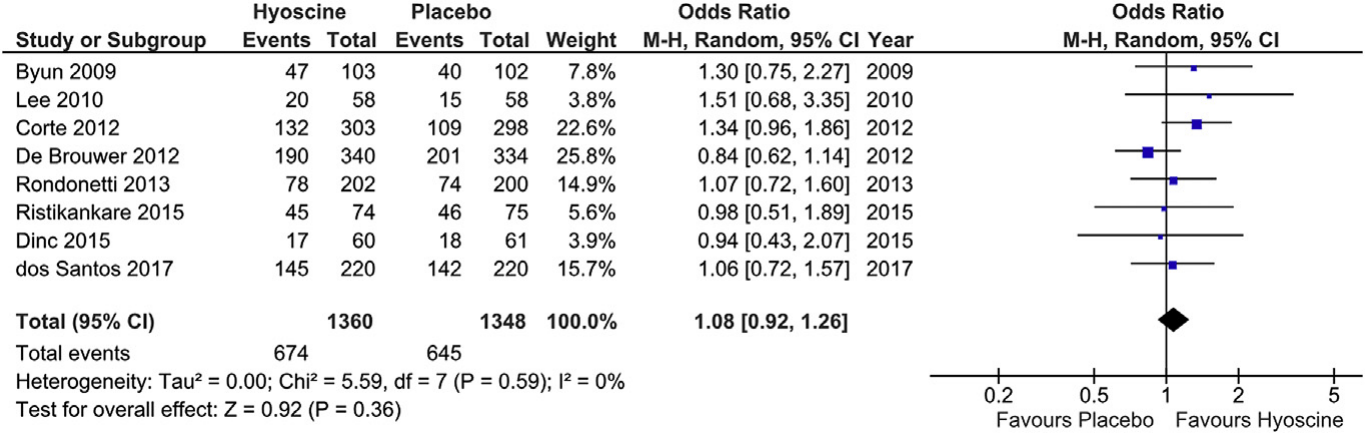
Forest plot of PDR.

PDR was considered the primary outcome in this meta-analysis. Eight RCTs assessed PDR [[10], [11], [12], [13], [14],[20], [21], [22]]. These trials included 2708 patients, of whom 1360 patients received Hyoscine Butylbromide and 1348 were allocated in the placebo group. 1319 patients were found to have polyps on colonoscopy, including 671 patients (49.3%) in the Hyoscine group and 648 patients (48%) in the placebo group. There was no significant difference between the groups (OR = 1.06, 95% CI: 0.90–1.23, P = 0.50) (Fig. 3). There was no significant heterogeneity (P = 0.54; I^2^ = 0%).

**Fig. 4:**
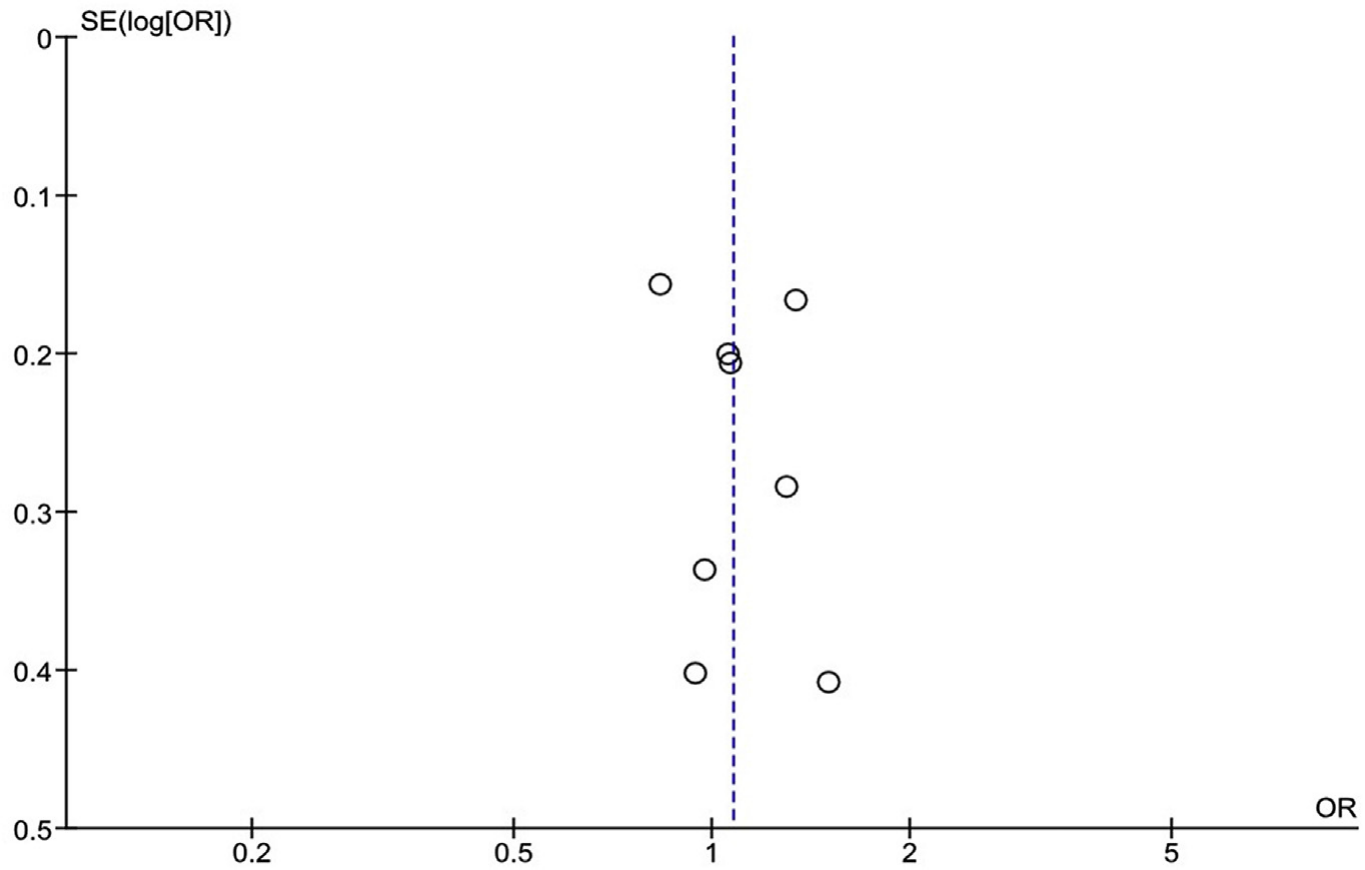
Funnel plot of the included studies.

Publication bias was evaluated by a funnel plots (Fig. 4). This revealed no significant publication bias in the meta-analysis.

### 3.2 Adenoma detection rate

**Fig. 5:**
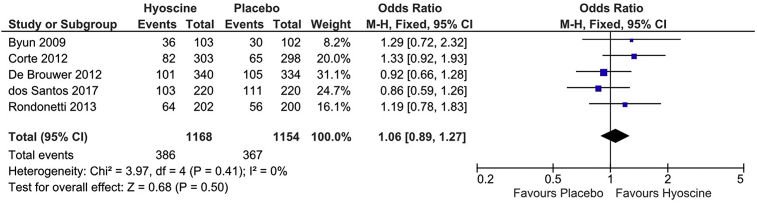
Forest plot of ADR.

Five RCTs assessed the ADR [10,11,13,14,21] (n = 2322). There was no significant difference in ADR between the Hyoscine and placebo groups (394/1168, 33.7% vs 359/1154, 31%; OR = 1.13;

95%CI: 0.95–1.35; P = 0.16) (Fig. 5). No heterogeneity was observed (P = 0.65, I^2^ = 0%).

**Fig. 6:**
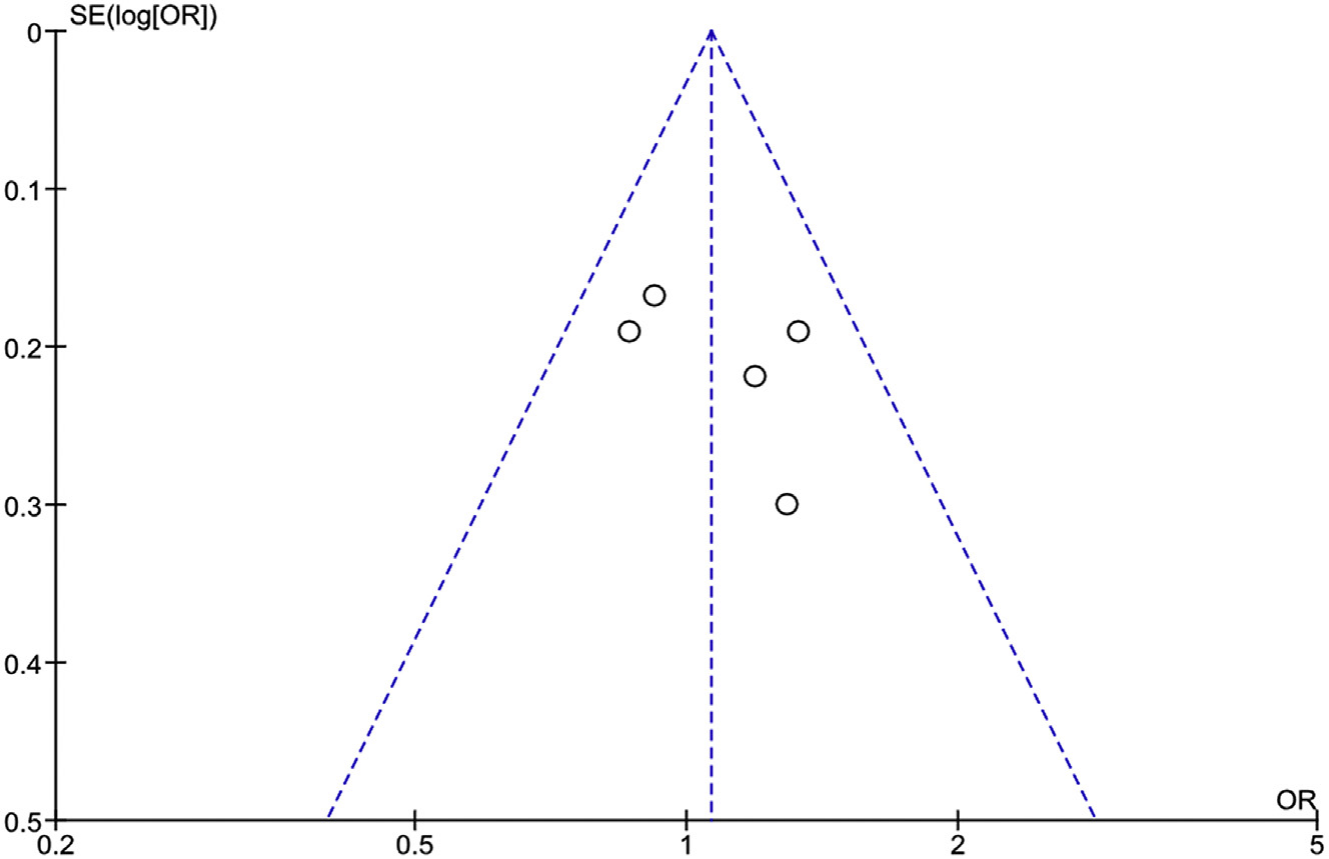
Funnel plot of the included studies for ADR.

Publication bias was evaluated by a funnel plot (Fig. 6). This revealed no significant publication bias in the meta-analysis.

## 4 Discussion

Polyps are premalignant lesions and removing them by colonoscopy is aimed to prevent malignant transformation [4]. ADR is the primary colonoscopy quality indicator [23]. Colonoscopy is the gold standard in detecting and removing polyps, however, it can potentially miss these polyps. Antispasmodic agents, like Hyoscine, have been suggested as means to improve the detection rate of polyps and adenomas by reducing spasms.

This study analyzed 2708 patients. Pooled data showed no statistically significant difference between the groups. Eight studies evaluated PDR [[10], [11], [12], [13], [14],[20], [21], [22]] and five studies assessed ADR [10,11,13,14,21]. This meta-analysis showed no significant effect of Hyoscine on PDR and ADR.

There are several strengths in this meta-analysis. It is the largest meta-analysis performed to date that incorporated 2708 patients. It included studies that were highly homogeneous (P = 0.54; I^2^ = 0%) compared to previous meta-analyses [[15], [16], [17]]. Most of the included studies in this meta-analysis were of high quality and minimal bias. Publication bias was not observed.

The findings of this paper do not support the routine use of Hyoscine. There is some evidence that Hyoscine can be, in fact, potentially counterproductive and might have a negative influence on detecting flat lesions as demonstrated by Rondonotti and colleagues [14]. This might be due to the reduction of the depth of the haustral folds caused by buscopan, which makes it more difficult to identify such lesions.

It is accepted that there are several confounding factors which will influence polyp detection at colonoscopy including endoscopist skill and bowel preparation. These are limitations on any study assessing the effects of Hyoscine on polyp detection rates and potentially could be compounded in any *meta*-anlysis. However, this research included only large scale studies with standardisation of endoscopist experience and bowel preparation to help limit these biases. It is also recognized that limiting the review to only English language reports is another limitation of this analysis.

The findings form this study are conflicting to the results of the recently published study from the English Bowel Cancer Screening Program [9], where the use of intravenous antispasmodic was associated with increased adenoma detection. This may be explained by a potential difference in endoscopists practice given that the use of Hyoscine was not randomized with different clinicians having different practices with a potential bias.

Given the standardisation of study data and number of cases reviewed, it is felt that this meta-analysis is able to conclude that the routine administration of Hyoscine Butylbromide does not improve polyp or adenoma detection rates.

## Ethical approval

NA.

## Sources of funding

NA.

## Author contribution

KH, OA AND PA COLLECTED THE DATA AND ANALYZED IT.

CE, LK AND LW REVISED THE MANUSCRIPT.

## Conflicts of interest

NA.

## Trial registry number

NA.

## Research registration number

Review Registry number of 478.

## Guarantor

KH, PA, OA.

## Provenance and peer review

Not commission, externally peer reviewed.
